# Dermoscopic aspect of verrucous epidermal nevi: new findings

**DOI:** 10.3906/sag-1811-27

**Published:** 2019-06-18

**Authors:** Ömer Faruk ELMAS, Necmettin AKDENİZ

**Affiliations:** 1 Department of Dermatology and Veneorology, Faculty of Medicine, Ahi Evran University, Kırşehir Turkey; 2 Department of Dermatology and Veneorology, Faculty of Medicine, İstanbul Medeniyet University, İstanbul Turkey

**Keywords:** Dermoscopy, large brown circles, verrucous epidermal nevus

## Abstract

**Background/aim:**

Verrucous epidermal nevi are cutaneous hamartomas with many clinical variants. Dermoscopic features of verrucous epidermal nevus have rarely been investigated. We aimed to identify dermoscopic findings of the entity which will facilitate the diagnostic process by reducing the use of invasive diagnostic methods.

**Materials and methods:**

The study included the patients with histopathologically approved verrucous epidermal nevus. Clinical, dermoscopic, and histopathological features of the patients were retrospectively reviewed and the findings identified were recorded. Dermoscopic examination was performed with a polarized-light handheld dermoscope with 10-fold magnification.

**Results:**

The most common dermoscopic features were thick brown circles, thick brown branched lines, and terminal hairs. The most common vessel pattern was dotted vessels. Branched thick brown lines, brown globules, brown dots forming lines, serpiginous brown dots, white and brown exophytic papillary structures, fine scale, thick adherent scale, and cerebriform structures were the other findings.

**Conclusion:**

We observed many vascular and nonvascular dermoscopic findings which were not described previously for the entity. Dermoscopic examination of the verrucous epidermal nevi may lead to more reliable clinical interpretation and thus may reduce the need for histopathological investigation.

## 1. Introduction

**Figure 1:**
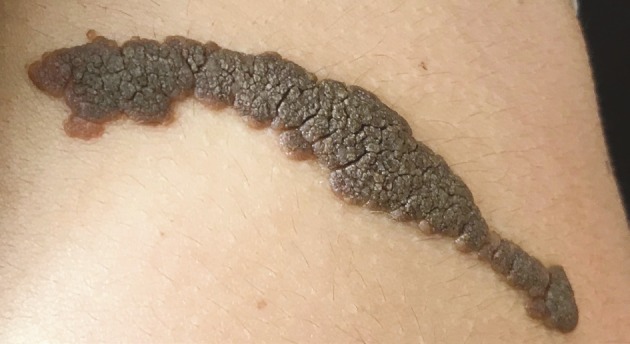
A verrucous epidermal nevus presented as an elevated solitary plaque localized on the abdominal skin of a young female patient.

**Figure 2:**
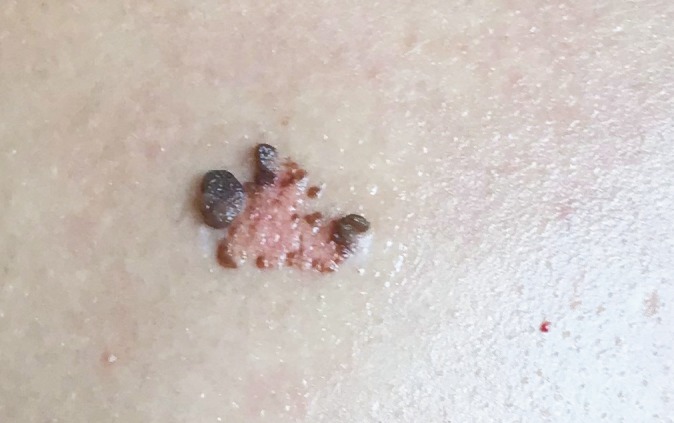
Another verrucous epidermal nevus characterized by well-circumscribed, grouped, exophytic structures localized on the back of a young male patient.

Epidermal nevi (EN) can be defined as cutaneous hamartomas with many clinical variants. Verrucous and well-circumscribed papillomatous lesions are the typical presentation of EN (Figures 1 and 2). The lesions can be seen anywhere and all components of the epidermis including keratinocytes, hair follicles, sebaceous glands, eccrine, and apocrine glands may be involved [1]. Verrucous epidermal nevus (VEN) is the most common variant of EN [2]. Epidermal nevus may be associated with some systemic abnormalities and this condition is known as epidermal nevus syndrome [3,4]. 

**Table 1:** Nonvascular dermoscopic findings of the patients with epidermal nevus.

Dermoscopic findings	Number and percentof the patient
Large brown circles	10 (50%)
Broad based, branched thick curved lines	1 (6.7%)
Thick brown lines	9 (45%)
Brown globules with surrounding white halo	4 (20%)
Whitish exophytic papillary structures	8 (40%)
Brown exophytic papillary structures	8 (40%)
Brown dots forming lines	1 (5%)
Serpiginous brown dots	1 (5%)
Cerebriform structures	1 (5%)
Comedo like openings	7 (35%)
Thick adherent scale	2 (10%)
Fine scale	2 (10%)
Terminal hairs	8 (40%)

**Table 2:** Vascular dermoscopic findings of the patients with epidermal nevus.

Dermoscopic findings	Number and percentof the patients
Dotted vessels	7 (35%)
Coiled Vessels	3 (15%)
Looped vessels	2 (10%)
Serpentine vessels	4 (20%)
Polymorphous vessel pattern	3 (15%)

Dermoscopy is a widely used noninvasive diagnostic tool in the diagnosis of many dermatological diseases. It is increasingly used for both melanocytic and nonmelanocytic skin lesions in daily dermatology practice. When reviewing the literature, it seems that there is just one original study investigating the dermoscopic features of EN, which included only 8 patients with VEN [2]. 

Here we aimed to identify the dermoscopic findings of VEN which will facilitate the diagnostic process by reducing the use of invasive diagnostic methods. 

## 2. Materials and methods

The study included the patients with histopathologically approved verrucous epidermal nevi between December 2017 and November 2018. The demographic, clinical, and dermoscopic features and the histopathological report of all the cases were retrospectively reviewed. Dermoscopic examination was performed with a polarized-light handheld dermoscope with 10-fold magnification (DermLite DL4; 3Gen, San Juan Capistrano, CA, USA). Dermoscopic photographing was done using a dermoscope-attached mobile phone with a high resolution camera (iPhone 7 Plus, Apple, California, USA). Descriptive statistical analysis was performed using the SPSS package program (SPSS Inc., Chicago, IL). All the procedures followed the Helsinki Declaration and the study was approved by the local clinical research ethics committee.

## 3. Results

A total of 20 patients were enrolled in the study. The mean age was 24 (range 11–42) and the majority was male (55%). Face was the most common localization (n = 9, 45%). The other localizations were trunk (n = 7, 35%) and neck (n = 4, 20%). Clinical examination revealed solitary papules or plaques in 12 (60%) patients (Figure 1) and well-circumscribed, grouped, exophytic structures (Figure 2) in 8 (40%) patients. 

The most common dermoscopic features were thick brown circles, thick brown branched lines, and terminal hairs. Dotted vessels were the most common vessel pattern. All the nonvascular and vascular dermoscopic features were detailed in Tables 1 and 2, respectively. 

When it comes to the histopathological features, all of the lesions showed typical histological features of verrucous epidermal nevus including hyperkeratosis, acanthosis, papillomatosis, and elongation of rete ridges. No histological signs of melanocytic nevi or malignant conditions were observed. 

## 4. Discussion

Recently, dermoscopy has become an important diagnostic tool in various dermatologic conditions. Dermoscopic features of many benign and malignant growths as well as many kinds of epidermal nevi like nevus sebaceous [5,6], hair follicle nevi [7], and nevus comedonicus [8] have been well described. However, the dermoscopic aspect of VEN has been the subject of only one original study in which Carbotti et al. [2] analyzed 8 patients with VEN. Here, we identified clinical and dermoscopic features of 20 cases of VEN.

**Figure 3:**
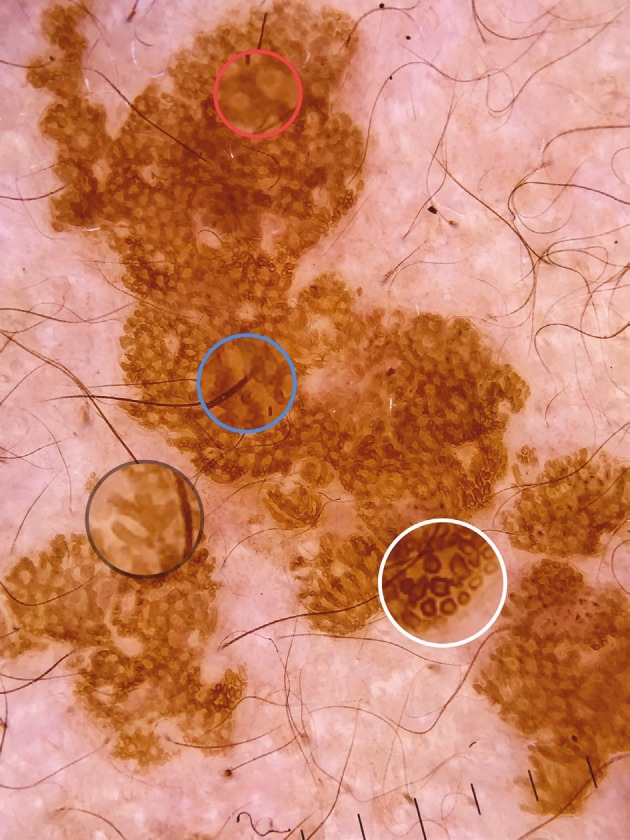
Large brown circles (white circle), thick brown branched lines (black circle), terminal hair (blue circle), brown globules (red circle).

**Figure 4:**
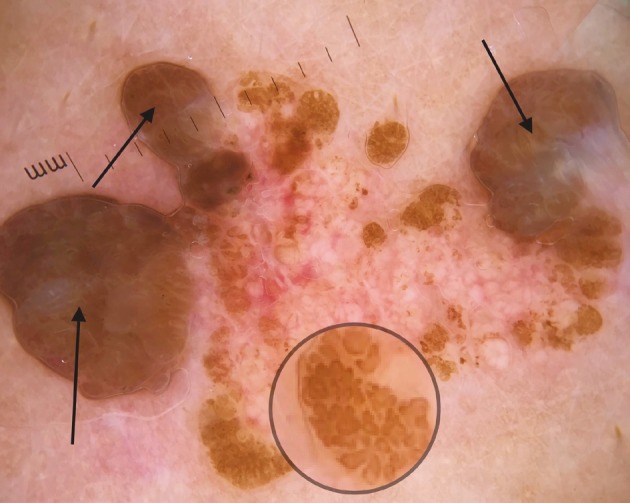
Brown exophytic structures (arrow), large brown circles (circle).

**Figure 5:**
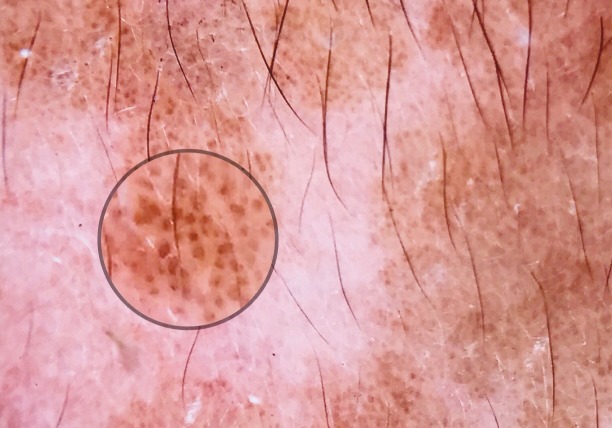
Brown globules (circle).

Carbotti et al. [2] reported that all the lesions of VEN showed large brown circles and absence of milia-like cysts, pigment network, and globules. They also noted that comedo-like openings were present in 3 out of 8 lesions [2]. In the present study, we observed that 10 (50%) out of 20 lesions showed large brown circles (Figures 3 and 4). The histological counterpart of the brown circles is thought to be peculiar disposition of pigmented keratinocytes surrounding the dermal papillae [2]. In the present study, comedo-like openings were present in 7 (35%) lesions. Comedo-like openings may correlate histologically to pseudohorn cysts in the epidermis opened to the surface of the lesion [9]. Brown globules (Figures 3 and 5) were present in 2 (13.3%) lesions and none of the lesions showed milia-like cysts and pigment network in our study. Brown globules histologically correspond to superficial dermal melanophages [10]. 

**Figure 6:**
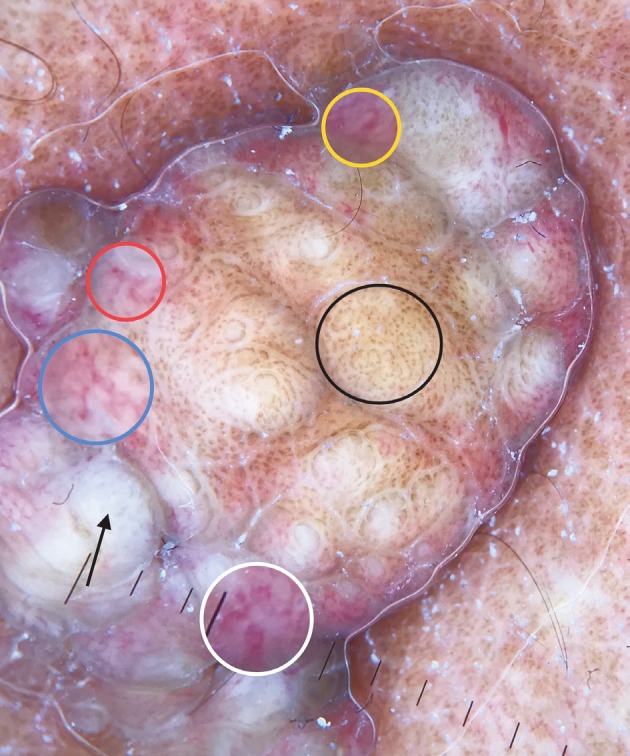
Brown dots forming lines and serpiginous brown dots (black circle), looped and serpentine vessels (yellow circle), serpentine and dotted vessels (red and blue circles), coiled vessels (white circle), white exophytic structures (arrow).

**Figure 7:**
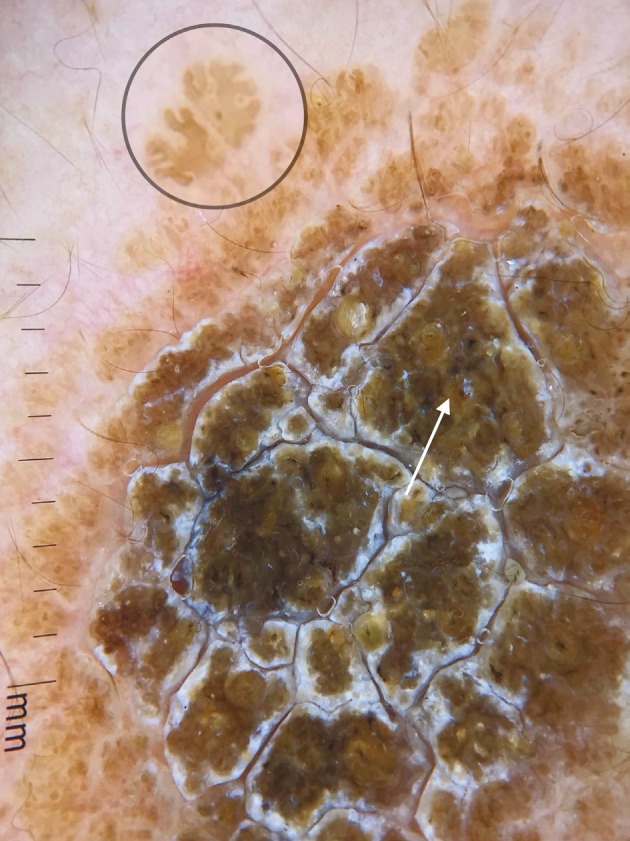
Thick adherent scale (arrow), broad based thick branched lines (circle).

**Figure 8:**
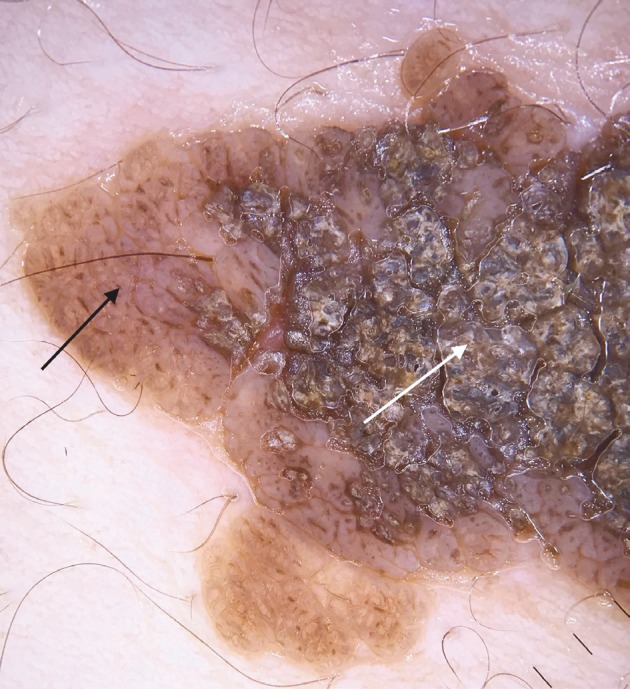
Thick adherent scale (white arrow), cerebriform structures (black arrow).

Here we identified 10 more nonvascular dermoscopic findings which were not described previously for VEN. These findings were branched thick brown lines (Figure 3), terminal hairs (Figure 3), brown globules (Figures 3 and 5), white and brown exophytic papillary structures (Figure 6), fine scale, thick adherent scale (Figure 7), broad based branched thick lines (Figure 7), brown dots forming lines (Figure 6), serpiginous brown dots (Figure 6), and cerebriform structures (Figure 8). 

Exophytic papillary areas are usually found in papillomatous dermal nevus [11]. Branched thick brown lines, comedo-like openings, and cerebriform structures are the typical dermoscopic features of seborrheic keratosis [12]. While the histological counterpart of thick brown lines is thought to be advanced epidermal acanthosis [10], cerebriform structures may correlate histologically to papillomatous surface of the epidermis [9]. 

When it comes to the vascular dermoscopic features, the most common vessel pattern was dotted vessels. Coiled, looped, serpentine, and polymorphous vessel patterns were also present in some lesions (Figure 6). Dotted, coiled, looped, and serpentine vessels can be seen in many benign and malignant conditions. Polymorphous vessel pattern means a combination of several vessel patterns and usually indicates a malignant tumor; however, it may also rarely be observed in benign conditions including eccrine poroma, pilomatrixoma, Clark’s nevus, and clear cell acanthoma [13,14]. In the present study, we observed polymorphous vessel pattern in 3 (20%) lesions (Figure 6).

The results of our study can be interpreted as follows;

1. All verrucous epidermal nevi may not show large brown circles dermoscopically. In the present study, half of the lesions did not exhibit this finding.

2. We observed 10 more nonvascular dermoscopic features which were not described previously for VEN. 

3. We also observed some vessel patterns which were not described previously for VEN.

4. VEN may share similar dermoscopic features with seborrheic keratosis and dermal nevus. 

In conclusion, we suggest that dermoscopic examination may lead to more reliable clinical interpretation and thus may reduce the need for histopathological investigation in patients with possible VEN. However, the entity may share similar dermoscopic characteristics with seborrheic keratosis and dermal nevus. In this context, although biopsy remains as the gold standard for definite diagnosis, no surgical intervention may be needed as almost all the differential diagnoses are benign conditions like seborrheic keratosis and dermal nevus. However, in case of the presence of polymorphous vascular pattern, which may be a clue to malignant growths, it would be reasonable to confirm the diagnosis histopathologically.

## References

[ref0] (2008). Benign epithelial tumors, hamartomas, and hyperplasias. Fitzpatrick’s Dermatology in General Medicine. 7th ed.

[ref1] (2016). Dermoscopy of verrucous epidermal nevus: large brown circles as a novel feature for diagnosis. International Journal of Dermatology.

[ref2] (2007). Epidermal nevus syndromes. Seminars in Cutaneous Medicine and Surgery.

[ref3] (2004). The epidermal nevus syndromes: multisystem disorders. Journal of the American Academy of Dermatology.

[ref4] (2009). Usefulness of dermatoscopy for the early diagnosis of sebaceous nevus and differentiation from aplasia cutis congenita. Clinical and Experimental Dermatology.

[ref5] (2008). The dermoscopic differential diagnosis of yellow lobular like structures. Archives of Dermatology.

[ref6] (2008). Hair follicle nevus – a dermoscopic approach. European Journal of Dermatology.

[ref7] (2013). Dermoscopy on nevus comedonicus: a case report and review of the literature. Postępy Dermatologii i Alergologii.

[ref8] (2017). Dermoscopy-pathology relationship in seborrheic keratosis. Journal of Dermatology.

[ref9] (2011). Dermatoscopy: an algorithmic method based on pattern analysis. Austria: Facultas Verlags und Buchhandels AG.

[ref10] (2009). Frequency, clinical and dermoscopic features of benign papillomatous melanocytic naevi (Unna type). British Journal of Dermatology.

[ref11] (2013). Dermoscopic features of facial pigmented skin lesions. ISRN Dermatology.

[ref12] (2015). Pigmented nodular melanoma: the predictive value of dermoscopic features using multivariate analysis. British Journal of Dermatology.

